# *TMEM173* variants and potential importance to human biology and disease

**DOI:** 10.1038/s41435-018-0029-9

**Published:** 2018-05-01

**Authors:** Seema Patel, Lei Jin

**Affiliations:** Division of Pulmonary, Critical Care and Sleep Medicine, Department of Medicine, University of Florida, Gainesville, FL 32610 USA

**Keywords:** Autoinflammatory syndrome, Genetic predisposition to disease

## Abstract

*TMEM173* gene encodes the protein STING (stimulator of interferon genes), a key player in host defense against pathogens. Mutations in the human *TMEM173* gene cause a life-threatening auto-inflammatory disease called SAVI (STING-associated vasculopathy with onset in infancy). Human STING is also a promising therapeutic target for cancers and infectious diseases. Recently, Aduro Biotech and Novartis announced a $250M-plus initiative to develop STING-targeting cancer immunotherapies. Thus, understanding the genetics of the human *TMEM173* gene is important for both basic and translational research. The human *TMEM173* gene has great heterogeneity and population stratification. *R232* of STING is the most common human *TMEM173* allele. However, >50% of Americans are not *R232/R232*. *HAQ* (R71H-G230A-R293Q) is the second most common human *TMEM173* allele. While *R232/R232* is the dominant *TMEM173* genotype in Europeans, *R232/HAQ* is the most common *TMEM173* genotype in East Asians. Importantly, recent studies suggested that *HAQ* and *H232* are likely loss-of-function *TMEM173* alleles. In all, ~30% of East Asians and ~10% of Europeans are *HAQ/HAQ*, *HAQ/H232*, or *H232/H232*. Here, we reviewed human *TMEM173* alleles, mutations and their potential impact on human health and medicine.

## Introduction

**Fig. 1:**
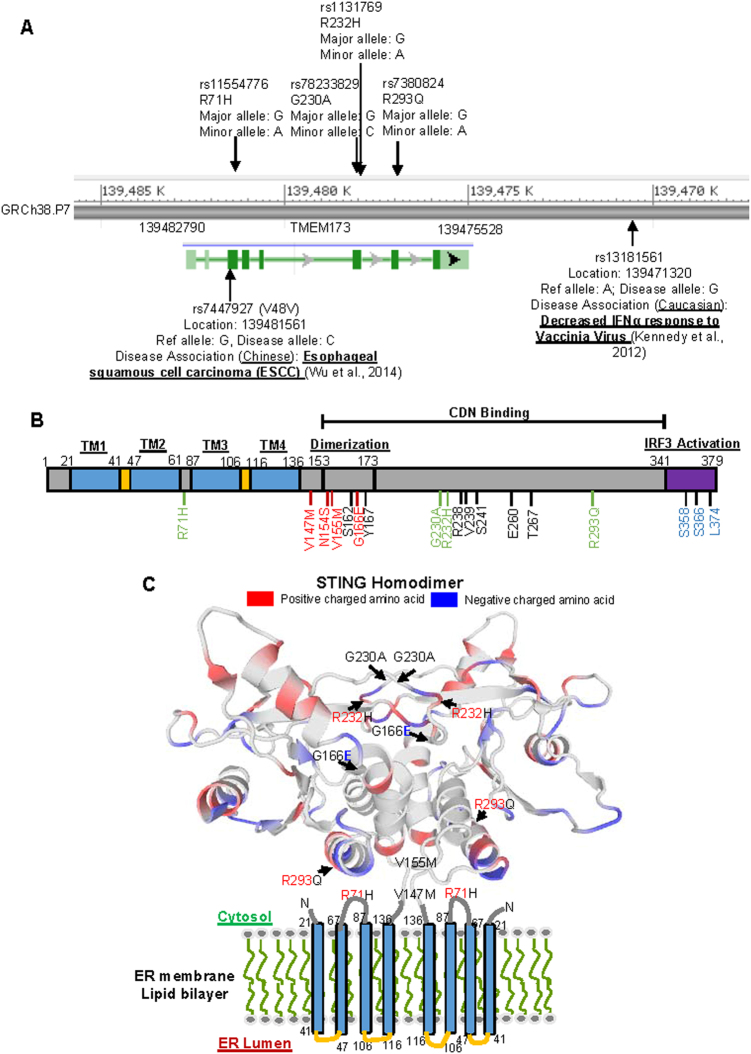
Human *TMEM173* gene and its associated diseases. **a** Cartoon illustrates human *TMEM173* gene (NCBI Reference Sequence: NC_000005.10). The common SNPs (R71H, G230A, R232H, and R293Q) are annotated along with the disease-associated SNPs (rs7447927 and rs13181561) identified in the published GWAS [28, 37]. **b** An annotation of the functional domains in the human STING protein [3–5, 23, 75, 76]. TM transmembrane region, CDN cyclic dinucleotide. Amino acids important for the CDN binding were in black. Common human STING variants were in green. Amino acids important for IRF3 activation were in blue. SAVI mutations were in red. **c** The structure of a human STING dimer anchored on the ER membrane [4, 23]. The cytoplasmic tails of the STING dimer form a butterfly-like binding pocket for CDN. The common STING variants G230A and R232H locate on the top (the lid region) of the binding pocket. The R293Q variant locates at the bottom of the pocket. The R71H variant locates in a predicted cytoplasmic loop facing the bottom of the binding pocket. The SAVI mutations V147M, N154S and V155M locate in the stem region of the binding pocket

DNA, including pathogen DNA from infection and mammalian DNA from damaged cells, stimulates STING (stimulator of interferon genes)-dependent type I interferon (IFN) production and promotes inflammation [1, 2]. STING is a four-transmembrane endoplasmic reticulum (ER) resident protein (Fig. 1) [1, 3]. It exists as a homodimer and undergoes a conformational change when binding to its ligands cyclic dinucleotides (CDNs) [3–5]. Activated STING homodimer then traffics through Golgi to the perinuclear region where it activates TANK Binding Kinase 1 (TBK1) leading to type I IFN production [1]. Human transmembrane protein 173 (*TMEM173*) *gene*, which encodes the STING protein, is a ~7kb-long gene at 5q31.2 (Fig. 1). STING is a critical player in host defense against pathogens, including HIV [6, 7], *Plasmodium* [8–10], and *Mycobacterium tuberculosis* [11–13]. STING also influences the development of autoimmune diseases [14, 15]. Last, pharmaceutical companies are developing STING-targeting immunotherapies [16–18]. The DNA-STING pathway has been reviewed extensively elsewhere [19, 20]. Here, we review the genetics of human *TMEM173* alleles, mutations and their potential impact on human health and medicine.

## Human population is highly heterogeneous for the *TMEM173* gene

The initial identified human STING has a Histidine at amino acid 232 (H232) [1, 2, 21]. We later found that *H232* was a minor allele [22]. The most common *TMEM173* allele in the human population has an Arginine at amino acid 232 (*R232*) [22]. In two cohorts of ~1000 Americans, ~45% are *R232/R232*, and only ~2% are *H232/H232* [22]. Notably, the *H232* allele is defective in response to CDNs in vitro [23, 24].

We also found that >50% of Americans contain at least one copy of non-*R232 TMEM173* allele, which suggested that the human population is highly heterogeneous for the *TMEM173* gene [22]. Common *TMEM173* alleles (>1% population frequency) are *R232*, *HAQ* (*R71H-G230A-R293Q*), *AQ* (*G230A-R293Q*), *Q293* and *H232* (Fig. 1) [22, 25]. *HAQ* is the second most common human *TMEM173* allele that contains triple non-synonymous single nucleotide polymorphisms (SNPs) (*R71H-G230A-R293Q*) [22]. Using B-cell lines derived from homozygous *HAQ/HAQ* individuals, we recently showed that *HAQ/HAQ* cells had extremely low STING protein expression and decreased *TMEM173* transcript compared to *R232/R232* cells [26]. Data from Genotype-Tissue Expression (GTEx) Portal (https://www.gtexportal.org/home/) showed that *HAQ* individuals had decreased *TMEM173* transcript in organs such as artery, skin, lung, and esophagus that are not B-cell dominant [26]. It is likely that *HAQ* is a null allele [26]. This notion was strengthened in vivo by studies in a knock-in *HAQ* mouse, which did not respond to CDNs [26]. Last, *HAQ* and *H232* alleles are in linkage disequilibrium with rs13181561 that is associated with >10-fold decrease of IFNα production to vaccinia virus stimulation in Europeans [27, 28]. It is worth noting that Sivick et al. [29] found no functional difference in PBMC among *HAQ* and *R232* individuals although their study had a small sample size with unknown ethnic origins.

The *HAQ* allele has significant population stratification. In Europeans, *R232/R232* is the most common genotype, while in East Asian, the dominant genotype is *HAQ/R232* [26]. Furthermore, ~16% of East Asians are *HAQ/HAQ* compared to ~3% in Europeans [26]. Interestingly, Africans have no *HAQ/HAQ* [26]. Instead, ~4% of Africans are *AQ/AQ*, which is absent in other ethnic populations [26]. Africans also have the *Q293* allele [26]. It is likely that *Q293* is the founder allele, where *AQ*, then *HAQ*, derived during the human migration out of Africa continent.

In summary, the human *TMEM173* gene has (i) great heterogeneity; (ii) significant population stratification; (iii) two possible loss-of-function alleles: *HAQ* and *H232*. In all, the *HAQ/HAQ*, *H232/HAQ*, and *H232/H232* genotypes account for ~30% of East Asians and ~10% of Europeans [26].

## *TMEM173* alleles in human health

STING is essential for host defense against DNA virus and some retroviruses [30]. Considering the vital role of viral infection in human evolution [31], it is surprising that such high percentages of the human population have possible loss-of-function *TMEM173* alleles. For example, Herpes simplex virus-1 (HSV-1), a DNA virus, has a high seroprevalence in the human population [32]. When infecting the central nervous system, HSV-1 causes herpes simplex encephalitis, the leading cause of viral encephalitis [33]. The initial study found that STING deficient mice were extremely susceptible to intravenous infection of HSV-1 [34]. So how *HAQ/HAQ*, *H232/H232*, and *HAQ/H232* individuals survived with HSV-1? Two recent studies may shed some light on it. They found that although STING^−/−^ mice were much more susceptible to intravenous HSV-1 infection than the WT mice, there were no difference in survival between the STING^−/−^ and WT mice following a mucosal HSV-1 infection route, which is a natural route of infection in humans [35, 36]. Furthermore, STING was not required for viral clearance and had a minimal effect on type I IFN production during the mucosal HSV-1 infection [35, 36]. Thus, the physiological role of STING in pathogen infection, especially in humans, need to be carefully evaluated.

Genome-wide association study (GWAS) identified two SNPs rs13181561 [28] and rs7447927 [37] within or near the human *TMEM173* gene (Fig. 1). In a search for genes associated with cytokine responses to vaccinia virus stimulation, Kennedy et al. [28], linked rs13181561 to decreased IFNα production in European (492 individuals), but not Africans (196 individuals) . rs13181561 is in linkage disequilibrium with *HAQ*, *H232* in Europeans [27], which indicates that these loss-of-function *TMEM173* alleles are associated with decreased IFNα production in response to vaccinia virus stimulation in Europeans.

In a joint analysis of three GWAS of esophageal squamous cell carcinoma (ESCC) in Chinese populations (5337 ESCC cases and 5787 controls), Wu et al. [37] found that rs7447927 was associated with ESCC in Chinese populations. rs7447927 is in linkage disequilibrium with rs13181561 [37], which is linked to *HAQ*, *H232* alleles. Thus *HAQ* and *H232* alleles are likely associated with susceptibility to the development of ESCC. It remains to be determined whether the loss of STING function in humans leads to the predisposition to ESCC.

## *TMEM173* mutations in SAVI (STING-associated vasculopathy with onset in infancy)

Activating mutations in the *TMEM173* gene lead to a newly classified rare auto-inflammatory disease call SAVI [38] (Table 1). It is an autosomal-dominant disease characterized by systemic inflammation, interstitial lung disease, cutaneous vasculitis, and recurrent bacterial infection [38, 39]. Both inherited, and de novo *TMEM173* mutations were found in SAVI patients (Table 1). SAVI with the de novo *TMEM173* mutations tended to have an early-onset (<8 weeks) and severe phenotype [38, 40], whereas familial *TMEM173* mutations had late-onset (teenager or adulthood) and milder clinical manifestations [39, 41]. For instance, SAVI patients with the inherited V155M mutation had a less severe disease penetration than patients with the de novo V155M mutation [38, 39, 42]. Jeremiah et al. [39], first found that the V155M mutation, at the steady state, localized mainly in the Golgi and in perinuclear vesicles of patient fibroblasts, which is a hallmark of the STING activation.

**Table 1:** A summary of identified activating *TMEM173* mutations in SAVI patien

*TMEM173*-activating mutations in SAVI patients			
Inherited *TMEM173*-activating mutations	Affected individuals	De novo *TMEM173*-activating mutations	Affected individuals
G166E	5	N154S	4
V155M	6	V155M	5
		V147M	2
		V147L	1
		C206Y	1
		R284G	1
		R281Q	1
		S102P-F279L	1

### SAVI as a unique interferonopathy with lung manifestation

SAVI is considered as a type I Interferonopathy that includes chronic atypical neutrophilic dermatosis with lipodystrophy and elevated temperature, Aicardi-Goutieres syndrome, and TREX1-SAMHD1-mediated familial chilblain lupus [40, 43, 44]. For example, familial SAVI mutations caused familial chilblain lupus [39, 41]. However, SAVI is unique because it is the only known type I Interferonopathy with pulmonary involvement [40, 43, 44]. In fact, all three reported fatalities from SAVI patients were due to the pulmonary complications [38, 40]. We showed that activating STING in the mouse lung by intranasal administration of CDNs, induced lung production of IFNγ and IFNλ but not IFNβ [45]. Interestingly, IFNγ^+^CD4^+^ T cells and serum IFNγ were markedly increased in a recent SAVI patient [46]. Notably, serum IL-18, a known IFNγ inducer, was also elevated in several SAVI patients [47]. Whether the increased IFNγ production contributes to the lung symptoms in SAVI patients is worth further investigation.

### Treating SAVI with JAK inhibitors

Current anti-inflammatory treatments corticosteroid, DMARDs, anti-TNF, steroids, anti-CD20, IVIG, were ineffective in SAVI patients [42, 47]. SAVI patients died of lung complication, and lung damage was irreversible [40, 47]. In fact, one SAVI patient died after double lung transplantation due to acute complications [40]. Thus, any SAVI treatment should result in improved lung function and prevent the irreversible lung damage.

Encouragingly, in a 2-year study with three SAVI children, ruxolitinib dramatically improved pulmonary function, resolved the cutaneous lesions and led to a better overall well-being of the patients [42, 48]. In a separate study, after a 3-month tofacitinib treatment, Seo et al. [46], saw an improved skin lesion in a SAVI teenager but the pulmonary defect remained. Eli Lilly is currently conducting a clinical trial (ClinicalTrials.gov number, NCT01724580) to examine the efficacy of baricitinib in SAVI patients.

Ruxolitinib and baricitinib are JAK1 and JAK2 inhibitors while tofacitinib is a JAK3 and to a lesser degree, JAK2 inhibitor. IFNα/β signals via JAK1/Tyk2 while IFNγ activates JAK1/JAK2. Thus, ruxolitinib and baricitinib are more suitable for treating SAVI than tofacitinib. Notably, baricitinib, at a high dose, also inhibits Tyk2, which mediates IL-10, IL-12/23, IL-6, and IL-4/13 signaling. Proper dosing may be important when treating SAVI patients with baricitinib.

### Loss-of-function human *TMEM173* allele as a natural inhibitor of SAVI

SAVI is caused by gain-of-function human *TMEM173* mutations [38] (Table 1). An intriguing question is whether the loss-of-function *TMEM173* alleles could serve as natural genetic inhibitors [49]. Cerboni et al. [49] found that in vitro, introducing *HAQ* into the V155M SAVI mutation (HAQ-V155M) relocated STING back to ER, restored T cell proliferation, and corrected NF-κB activation . Recently, a de novo SAVI patient was identified in a *HAQ* family [46]. In this case, the activating *TMEM173* mutation acts in trans with the *HAQ* allele [46]. The patient exhibited SAVI symptoms but with a late-onset (3 years) [46]. Thus, the presence of the *HAQ* allele could be advantageous to SAVI patients.

## *TMEM173* mutations in human cancers

STING can promote [50, 51] or suppress [52, 53] tumorigenesis in mice. Xia et al., sequenced the *TMEM173* gene in 11 human colon cancer cell lines and 11 human melanoma-derived cell lines [54, 55]. No somatic *TMEM173* mutations were found, although 2 out of the 11 colon cancer lines and 7 out of the 11 melanoma lines carried the *HAQ* allele [54, 55]. Data from the COSMIC (The Catalogue of Somatic Mutations in Cancer) confirmed that somatic human *TMEM173* mutation is rare in cancers [56, 57]. Out of 30,710 primary human cancer samples, only 43 samples have somatic *TMEM173* mutations, a mutation rate of 0.11% [56, 57] (Table 2).

**Table 2:** Somatic *TMEM173* mutations in primary human cancer tissues

Somatic *TMEM173* mutations in cancer subtypes				
Tissue	Samples with mutations	Tested samples	Mutation	Mutation rate (%)
Skin-face basal cell carcinoma	1	5	G251E	20.00
Skin-head neck squamous cell carcinoma	2	39	S53F, L285I	5.13
Skin-basal cell carcinoma	2	49	P92L, [R94C,Y274D]^a^	4.08
Lung-right lower lobe adenocarcinoma	1	45	R284M	2.22
NS-malignant melanoma	2	101	R232Y, G192S	1.98
Esophagus-lower third squamous cell carcinoma	1	54	N131fs*13	1.85
Large intestine-cecum adenocarcinoma	2	125	V85fs*46, R253Q	1.60
Soft tissue-rhabdomyosarcoma	1	81	N183S	1.21
Stomach-intestinal adenocarcinoma	1	85	T356M	1.18
Urinary tract-bladder transitional cell carcinoma	1	114	H50Q	0.88
Large intestine-adenocarcinoma	3	384	G35E,R76G,L285P	0.78
Endometrium-endometrioid carcinoma	4	548	R180Q,R197Q, Q276P,R375C	0.73
Large intestine-colon adenocarcinoma	4	715	P40S,R197W,R310H,G344C	0.70
Liver-neoplasm	1	162	A18D	0.62
Upper aerodigestive tract-mouth squamous cell carcinoma	1	221	L136P	0.45
Liver-hepatocellular carcinoma	4	921	T376K,R375C,G344D,V329F	0.43
Skin-malignant melanoma	3	818	W82R, L202F, P371L	0.37
Urinary tract-bladder carcinoma	2	554	H50Q,D205N	0.36
Lung-squamous cell carcinoma	2	655	L133F,E282*stop	0.31
Kidney-papillary renal cell carcinoma	1	335	R375H	0.30
Liver-carcinoma	1	725	F378L	0.14
Lung-adenocarcinoma	1	772	S4C	0.13
Kidney-clear cell renal cell carcinoma	1	865	H74Y	0.12
Breast-carcinoma	1	1263	D210N	0.08

Data were extracted from the COSMIC database (The Catalogue of Somatic Mutations in Cancer) [56, 57]

^a^These two mutations were found in the same sample

Among the somatic human *TMEM173* mutations identified (Table 2), R284M is an activating *TMEM173* mutation [58]. This mutation was found in a lung-right lower lobe adenocarcinoma (Table 2) [56]. Interestingly, a similar *TMEM173*-activating mutant, R284G, was recently found in a SAVI patient [59]. This patient had recurrent bacterial infections in the upper respiratory tract but never had systemic markers of inflammation [59]. Her lung function was also normal [59]. Thus, the activating R284M mutation in the lung cancer sample is likely not causative. In conclusion, somatic *TMEM173* gene mutation is rare in human cancers and does not seem to play a major role in tumorigenesis.

## *TMEM173* gene expression in human cancers

Decreased STING expression was observed in some human melanoma cell lines and tissues [55, 60]. In COSMIC database, however, out of 9110 primary human cancer tissues, 313 samples (3.44%) have overexpressed *TMEM173* gene expression (*Z*-score > 2) compared to the 17 samples (0.19%) that have underexpressed *TMEM173* gene (*Z*-score < −2) [56, 57]. Samples from lung cancers, brain cancers, and kidney cancers have the highest rate of *TMEM173* gene overexpression [56, 57].

Why do cancer samples have overexpressed *TMEM173* gene? The vast majority of those cancer samples did not have somatic *TMEM173* mutations. Wang et al. [61], recently reported that c-Myc binds to the −124 to 1 bp of the human *TMEM173* gene and promotes its transcription. c-Myc is activated in many cancers. It is tempting to suggest that *TMEM173* overexpression in human cancer samples is driven by the activated c-Myc. Whether the overexpressed *TMEM173* gene in human cancer samples contributes to tumorigenesis, remains to be determined.

## *TMEM173* alleles in human medicine

STING is a promising therapeutic target for cancer immunotherapies [16–18, 62]. Pneumovax23® vaccine efficacy depends on STING in mice [26, 63]. STING may also contribute to the efficacy of the radiotherapy [16] and chemotherapy [55, 64] likely due to DNA released during these treatments. Using a mouse model of the human *HAQ* allele, we found that Pneumovax23® was ineffective in the HAQ mouse [26]. Furthermore, CDNs lost its adjuvant activity in the HAQ mouse [26]. The low expression of STING in the *HAQ/HAQ* individuals likely will affect the efficacy of STING-targeting cancer immunotherapies.

On the other hand, increased STING expression in primary human cancer samples provides a rationale for targeting STING for cancer treatments. STING/MPYS was initially identified as an apoptotic molecule mediating anti-MHC II mAb induced cell death in mouse B-cell lymphomas [21]. Recently, several studies showed that direct activation of STING by CDNs could kill tumors [65, 66]. Thus, tumors with increased STING expression may be killed directly by the activation of STING/MPYS-mediated cell death pathway. On the other hand, activation of the STING pathway in DCs promotes DCs maturation [67, 68]. These mature DCs can initiate CD8^+^ T cells-mediated cytotoxic response and generate memory response to prevent cancer relapse [17, 18, 69–73].

## Future directions

Since its discovery in 2008, most of our knowledge on STING has been from mouse studies. These studies have established a critical role of STING in infectious diseases and autoimmune diseases. The identification of SAVI mutations in 2014 established an unambiguous role of the *TMEM173* gene in the human auto-inflammatory disease. However, the role of *TMEM173* in human infectious diseases remains to be determined. A recent study done in two independent European cohorts (150 patients and 188 controls) found that the population frequency of *HAQ* increased in human Legionnaires’ disease patients as compared to healthy controls, which suggested that *HAQ* carriers may be more susceptible to Legionnaires’ disease than the *R232* carriers [74]. Questions remain whether *HAQ* affects individual’s susceptibility/resistance to other human pathogens such as HIV, *Plasmodium*, or *Mycobacterial tuberculosis*. Meanwhile, a GWAS study revealed an association between the *TMEM173* gene and ESCC [37]. It remains to be determined whether the *HAQ* and *H232* alleles associated with ESCC are causative, which will greatly enhance the mechanistic understanding of this disease.

The animal and in vitro studies indicated that STING might influence the efficacy of some human medicines [16, 26, 64]. It will be beneficial to determine whether the *HAQ* and *H232* alleles cause decreased efficacy for Pneumovax23®, chemotherapy, and radiotherapy so that right patients can be benefited from these medications.

Lastly, the *HAQ* allele is common in East Asians and rare in Africans [26]. The *H232* allele, however, does not show such population stratification [26]. Thus, the environmental pressure selecting for the *HAQ* allele seems to be different from the *H232* allele. Determining the environmental factor(s) selecting for these loss-of-function *TMEM173* alleles will help us understand the physiological function of the human *TMEM173* gene.
